# Torsion of Arteries for the Arrest of Hæmorrhage1Read before the National Association of Railway Surgeons, at St. Louis, Mo., May 2, 1889.

**Published:** 1889-06

**Authors:** J. B. Murdoch

**Affiliations:** Pittsburgh, Penna


### TORSION OF ARTERIES FOR THE ARREST OF HÆMORRHAGE.[1]

BY DR. J. B. MURDOCH, PITTSBURGH, PENNA.

Upon no subject has our profession been more conservative than upon the manner of arresting hæmorrhage after wounds of arteries and veins. Since the time of Celsus, notwithstanding the numerous methods which have been proposed for this purpose, but two, viz., the actual cautery and the ligature, have received the indorsement of the profession. But if the profession has been slow to endorse new methods its confidence once gained has not been easily shaken. This is illustrated by the tenacity with which surgeons adhered to the actual cautery after the discovery of the ligature.

The efforts made by Sir James Simpson, of Edinburgh, to substitute acupressure, and the still more recent endeavor of Dr. S. F. Spier, of Brooklyn, to substitute constriction for ligation have most signally failed. The same statement may be made also in regard to torsion as a means of arresting arterial hæmorrhage. It has not received the support of the profession to any great extent, but unlike the other rivals of the ligature it has had champions for hundreds of years, and still holds a place as a valuable means of arresting hæmorrhage. This subject has received but little attention by modern surgeons. The twisting of an artery to arrest bleeding is of ancient origin. It is spoken of by Celsus. A fact often observed that an arm or leg may be torn from the body with the loss of only a few drops of blood no doubt suggested the method. It has been advocated by such surgeons as Amussat, Dieffenbach, Schroeder, and Syme. But the credit of bringing it prominently before the profession and establishing its efficiency is due to Bryant, the present distinguished Surgeon of Guy's Hospital, London. At this hospital the ligature is seldom used, torsion being chiefly relied upon. Mr. Bryant tells us in the last edition of his Surgery that in two hundred consecutive amputations of the thigh, leg, arm, and forearm all of the arteries were twisted, one hundred and ten of them being the femoral artery, and that in no case was there secondary hæmorrhage.

Mr. Bryant says: "The physiological arguments in favor of torsion are very great, and the practical advantages seem to be no less. After seven years' experience in its practice, applied to vessels of all sizes, the femoral being the largest, I have had no mishap. I have observed that wounds have united more rapidly and kindly, primary union being the rule. There has been less constitutional disturbance after operation, and consequently less liability to traumatic fever, pyæmia, and other complications such as we are all too familiar with in the practice of surgery. I have had stumps heal in a week

---

[1] Read before the National Association of Railway Surgeons, at St. Louis, Mo., May 2, 1889.

and the patient up in two weeks; without one single drawback, rapid and uninterrupted convalescence following the operation."

Having given this experience of Mr. Bryant, I desire now to give my own as observed at the Western Pennsylvania Hospital of Pittsburgh. At this hospital torsion is almost exclusively relied upon to check the hæmorrhage from wounded arteries or veins, whether the wound be produced by the surgeon's knife or otherwise. My experience with torsion, as a hæmostatic, dates back to the year 1872, when I became a member of the hospital staff. My colleague had, previous to my connection with the hospital staff, been twisting arteries as large as the radial and ulnar. The facility with which this was done, and the fact that the wounds healed kindly and without secondary hæmorrhage, induced me to follow their example, at first timidly, but with success came confidence. Having been successful in the amputation of a forearm with no untoward result, I ventured next to twist the brachial after the amputation of an arm; soon after this the axillary and then the popliteal, and finally the femoral. And now for the past fourteen years torsion for the arrest of hæmorrhage after all surgical operations has been the recognized, and almost the only method resorted to at this hospital. It is to be regretted that records have not been kept of the number of large arteries which have been twisted to arrest the hæmorrhage.

The following is a table showing the number of arteries divided in cases of amputation where torsion has been resorted to for the arrest of hæmorrhage at the Western Pennsylvania Hospital:

| | | |
|---|---|---|
| Femoral | 95 | times. |
| Popliteal | 14 | " |
| Axillary | 15 | " |
| Anterior tibial | 276 | " |
| Posterior " | 276 | " |
| Brachial | 62 | " |
| Radial | 40 | times. |
| Ulnar | 40 | " |

The manner in which the torsion was applied in these cases is the same as described by Mr. Bryant in his Surgery:

"A good pair of forceps is required which will hold the end of the artery firmly, that has no lateral motion, and with serrations blunt enough to obviate any laceration or cutting of the parts seized by the blades. The vessel should then be drawn out as in the application of the ligature and three or four sharp rotations of the forceps made. In large arteries, such as the femoral, the rotation should be repeated till the sense of resistance has ceased. The ends should not be twisted off. In small arteries the number of rotations is of no importance and their ends may be twisted off or not, as may be preferred. In all of the cases mentioned in the above table torsion of the arteries and veins was the method resorted to to control hæmorrhage.

In addition to these cases, of which we have a record, the method of torsion has been the one resorted to in all other surgical operations performed during this period, such as amputations of the female breast, the removal of tumors, the excision of joints, etc. It is within bounds to say that torsion has been resorted to at this hospital in thousands of cases without any mishap. We have had no case of secondary hæmorrhage which could fairly be attributed to the method of controlling the hæmorrhage.

The advantages of torsion as compared with ligation are:

1. The greater facility with which it can be applied.

I am fully aware that this proposition is disputed, but to those who are familiar with both methods there can be no doubt that torsion is the easier of the two. For the ligation of an artery an assistant is required to seize the vessel and draw it out while the ligature is applied. For torsion the surgeon requires no assistant. The vessel must be seized by the forceps in either case. In torsion it only requires three or four turns of the forceps to complete the process which can be accomplished in as many seconds. When a ligature is applied, let the operator be ever so skillful, the thread may break or slip off the vessel, but if neither of these accidents occur, the process can not be accomplished in anything like the same time.

2. Torsion is a safer method, being less liable to be followed by secondary hæmorrhage.

This proposition has been absolutely proven by the experience in the use of torsion at Guy's Hospital, London, and I have now given additional proof by the experience given in this paper.

3. Healing is facilitated because the wound is free from any irritating or foreign body.

This proposition is so plain that it should not require an argument. It was true before the antiseptic treatment of wounds had come into such general use, but is doubly so now. The catgut ligature is no doubt a safer ligature than the silk, for it does not require an ulcerative process for its discharge, and when this ligature has been made thoroughly antiseptic, it is no doubt the best. But a ligature rendered thoroughly antiseptic is not always at hand, and those surgeons who have had most experience with the antiseptic treament of wounds will, I think, be the first to admit that in spite of their most careful attention septic germs are often introduced into the wounds by means of the ligature. Even after over-protection in preparation and preservation, the handling of a ligature in its application is a frequent source of infection.

But there are other objections to its use. The catgut ligature may dissolve before the artery has become closed by the natural hæmostatic process, or it may unbind. Both of these accidents have been the frequent cause of secondary hæmorrhage.

On a recent visit to some of the principal hospitals of New York City, where the operators and assistants possessed the greatest skill, I was not surprised to see that in many instances a ligature broke, and in other cases slipped off the vessels before they were secured. This was to me exceedingly annoying to witness, when I knew that the vessels could have been so easily twisted while they were in the grasp of the forceps. When the question was asked one of these operators, a distinguished surgeon, Why don't you resort to torsion? the reply was, "we are afraid to trust it." This answer might have been given with equal force by Richard Wiseman in the 17th century when asked why he did not resort to the ligature instead of the red-hot iron.

In a matter so important as the arrest of arterial hæmorrhage, it is proper that surgeons should be conservative, but there is such a thing as pushing conservatism too far. In the torsion of arteries I claim we have an improvement upon ligation; its claims for recognition rest upon physiological arguments which can not be shaken, and its reliability as a hæmostatic has been proven by abundant experience.

